# Life‐course socioeconomic position and changes in cognitive function in Hispanic/Latino adults. Results from the Hispanic Community Health Study/Study of Latinos (HCHS/SOL)

**DOI:** 10.1002/alz.085600

**Published:** 2025-01-09

**Authors:** Paola Filigrana, Jee‐Young Moon, Richard B. Lipton, Wassim Tarraf, Larissa Aviles‐Santa, Martha L Daviglus, Linda C Gallo, Krista M Perreira, Ariana M Stickel, Fernando Daniel Testai, Bharat Thyagarajan, Hector M González, Carmen R Isasi

**Affiliations:** ^1^ Albert Einstein College of Medicine, Bronx, NY USA; ^2^ Department of Neurology, Albert Einstein College of Medicine, Bronx, NY USA; ^3^ Wayne State University, Detroit, MI USA; ^4^ National Institute of Health, Bethesda, MD USA; ^5^ University of Illinois at Chicago, Chicago, IL USA; ^6^ San Diego State University, San Diego, CA USA; ^7^ University of North Carolina, Chapel Hill, NC USA; ^8^ University of Illinois at Chicago, College of Medicine, Chicago, IL USA; ^9^ University of Minnesota, Minneapolis, MN USA; ^10^ University of California, San Diego, La Jolla, CA USA

## Abstract

**Background:**

Socioeconomic disadvantage at different life‐course stages has been associated with later life cognitive impairment. However, its association with changes in cognitive function needs to be further elucidated. We assessed the association between socioeconomic position (SEP) throughout the life‐course and cognitive function change in middle‐aged and older Hispanic/Latino adults.

**Method:**

We used data from the Hispanic Community Health Study/Study of Latinos (HCHS/SOL), a multicenter community‐based cohort of Latinos in the US (n = 6351; age range 45‐74) and a follow up visit conducted 7 years later by the SOL‐INCA ancillary study. Childhood SEP was determined using parental education (<high school [HS], HS, >HS). An index combining participants’ education, household income, occupation, and assets determined midlife SEP (low, middle, high SEP). Using dichotomized childhood and midlife SEP, we classified participants into socioeconomic mobility categories (stable low or high SEP, upward or downward mobility). Cognitive function changes (7‐years change) were ascertained as the difference in test scores between visits. Cognitive function assessments included the Six‐Item Screener, Brief‐Spanish English Verbal Learning Test Sum and Recall, Controlled Oral Word Association, and the Digit Symbol Substitution Test. Cognitive measures were z‐score standardized and a global cognition (GC) score was created using confirmatory factor analysis. Survey linear regression models were performed accounting for demographic, behavioral, and clinical covariates, baseline cognitive function, and years from baseline.

**Result:**

Higher midlife SEP was associated with a slower decline in cognitive function in later life (b for GC middle vs. low SEP: 0.12, 95% CI: 0.05, 0.19; and high vs. low SEP: 0.26, 95% CI: 0.15, 0.35). Upward socioeconomic mobility vs. stable low SEP (GC b: 0.29, 95% CI: 0.20, 0.39) and stable high SEP vs. stable low (GC b: 0.43, 95% CI: 0.32, 0.54) were also associated with slower cognitive decline. Results were similar for each individual cognitive test. We did not find associations between childhood SEP and changes in cognitive function.

**Conclusion:**

This study highlights the benefits on cognitive aging of advantageous SEP over the life‐course. Having a higher midlife SEP, upward socioeconomic mobility, or a stable high SEP over the life‐course showed protection for cognitive decline in adulthood.

